# Classification Systems of Cleft Lip, Alveolus and Palate: Results of an International Survey

**DOI:** 10.1177/10556656211057368

**Published:** 2021-11-23

**Authors:** Ruben Houkes, Johannes Smit, Peter Mossey, Peter Don Griot, Martin Persson, Amanda Neville, Edwin Ongkosuwito, Tom Sitzman, Corstiaan Breugem

**Affiliations:** 1 Department of Plastic Surgery, 522567 Amsterdam University Medical Center, Emma Children’s Hospital, AZ, Amsterdam, the Netherlands; 2 Department of Dentistry, University of Dundee Dental Hospital & School, Dundee, Scotland, UK; 3 Faculty of Health Sciences, 4342 Kristianstad University, Kristianstad, Sweden; 4 Center for Clinical and Epidemiological Research, 60221 University of Ferrara, Ferrara, Italy; 5 Department of Dentistry - Orthodontics and Craniofacial Biology, 6034 Radboud University Medical Center, Nijmegen, the Netherlands; 6 Division of Plastic Surgery, Phoenix Children's Hospital, Phoenix, AZ, USA

**Keywords:** Classification system, cleft lip, cleft palate, orofacial cleft, survey

## Abstract

**Objective:**

This study aimed to identify commonly used classification systems by cleft providers around the world, including the perceived indications and limitations of each system.

**Design:**

A cross-sectional survey.

**Participants:**

A total of 197 registrants from three international cleft/craniofacial meetings.

**Interventions:**

Participants were sent a web-based questionnaire concerning cleft classification systems.

**Main Outcome Measures:**

Frequency of commonly used classification systems, their perceived indications and limitations.

**Results:**

A total of 197 respondents from 166 different centers completed the questionnaire. Healthcare professionals from all disciplines responded, with the most frequent respondents being plastic surgeons (38.1%), maxillofacial surgeons (28.4%) and orthodontists (23.9%). Eighteen different classification systems were in use. The most frequently used systems were the International Statistical Classification of Diseases and Related Health Problems (ICD-10) (35.5%), LAHSHAL (34.0%), and Veau (32.5%) classification systems. Most respondents (32.5%) indicated that anatomical and morphological characteristics are essential components of a classification system. However, respondents indicated that their current classification systems lacked sufficient description of cleft extension and severity.

**Conclusions:**

Great variety in the use of classification systems exists among craniofacial specialists internationally. The results recommend the usage of the LAHSHAL classification of OFCs, due to its comprehensiveness, relatively high implementation rate globally, convenience of usage and complementarity with the ICD-10 system. Moreover, it can overcome deficiencies inextricably linked to ICD-10, such as incapacity to describe laterality and clefts of the alveolus. More international exposure to the merits of using the LAHSHAL classification system would be highly recommended.

## Introduction

Orofacial clefts (OFCs) are the most common type of craniofacial anomaly, occurring in approximately 1:700 live births (Calzolari et al., 2007; Mossey and Modell, 2012; Mai et al., 2019; World Health Organization, 2021a). OFCs can have a profound impact on a person's quality of life, which can lead to problems with feeding, speech (eg velopharyngeal insufficiency), hearing (eg recurring otitis media), dentition (eg tooth decay), neurodevelopmental disorders, psychological aspects and socialization (Gomes et al., 2009; Wehby and Cassell, 2010; Smallridge et al., 2015; Waylen et al., 2015; Tillman et al., 2018). The physiological severity of these problems is not correlated with the severity and location of the cleft. However, accurate cleft classification is essential for predicting treatment need, planning clinical interventions and evaluating treatment outcomes. Consequently, classifying clefts in a clear, easy to understand and concise manner is a key element of cleft care.

Cleft classification also plays an important role in studying the epidemiology of OFCs. OFCs are etiologically heterogeneous, and untangling the interactions of environmental and genetic risk factors leading to OFCs requires accurate classification of cleft type (Dixon et al., 2011). More specific classification systems enable greater subgrouping of clefts, which can facilitate identification of the causes and biologic mechanisms for OFCs. The value of cleft classification to epidemiology is perhaps best illustrated by the different recurrence risks for cleft lip alveolus, cleft lip alveolus and palate and cleft palate, which is a key difference used in genetic counseling (Sivertsen et al., 2008; Grosen et al., 2010). A standardized, accurate phenotypic classification of clefts is thus crucial to understand the epidemiology of OFCs.

During the history of cleft care, many classification systems have been developed and implemented. New classification systems have often been developed to classify clefts more accurately or to overcome limitations of existing systems. For example, the Veau classification system was developed as a response to the Brophy classification system, since Brophy was considered too complex and impractical (Allori et al., 2017). Another classification system is that of Fogh-Andersen, which was designed from an embryological point of view (Fogh-Andersen, 1971; Allori et al., 2017). Fogh-Andersen found that the alveolar process as a marking point was too arbitrary and suggested to use the incisive foramen instead. In 1989, the LAHSHAL recording system or registration system was designed for paraphrasing cleft lip and palate in which a distinction could be made between unilateral and bilateral clefts and to describe the extent of clefting (ie total vs subtotal clefts) (Kriens, 1989). While each of these classification systems provided value, none achieved universal acceptance among providers.

More recently, the International Statistical Classification of Diseases and Related Health Problems (ICD) system has been promoted for both epidemiology and reimbursement (World Health Organization, 2021b). The ICD system is used in the WHO Global Burden of Disease (GBD) project, which has made reporting by ICD codes required for most governmental health registries (Truelsen et al., 2015; World Health Organization, 2021c). Healthcare payers in both Europe and North America have also mandated use of the ICD system for reimbursements. These two forces have led to broad implementation of the ICD system, despite concerns with its lack of specificity, mutual exclusivity, and comprehensiveness for classifying OFCs.

It is prudent to make a difference between registration and classification of clefts. For example, the ICD-10 classification, although described as a “classification”, is actually a registration process for coding and categorization of disease as opposed to being designed for the primary purpose of classification *per se*. This article makes no particular distinction between registration and classification systems.

As a result of great variety in possible classification systems, craniofacial specialists often use different classification systems. This hampers mutual understanding, communication and treatment outcome comparison among specialists and prevents the development of a uniform prevalence/incidence system. The aim of this article is to describe which classification systems are used globally, including their perceived indications and limitations. Furthermore, it aims to make a recommendation on which system is most suitable for global implementation so development of a global prevalence system and improvement of scientific data comparison is achievable.

## Methods

This study was a cross-sectional survey. A questionnaire was sent to 2140 registrants of the 2015 Cleft Craniofacial meeting in Gothenburg, Sweden, the 2017 13^th^ International Cleft Meeting in Chennai, India, and the 2019 European Cleft Palate Craniofacial Association (ECPCA) meeting in Utrecht, the Netherlands. The questionnaire was also sent to American Cleft Palate-Craniofacial Association (ACPA) members through the ACPA member portal and to members of the European Cooperation in Science and Technology (COST) Action and members of the European Cleft and Craniofacial Initiative for Equality in Care (ECCE).

The questionnaire comprised a combination of 20 multiple choice and open-ended questions investigating the use of cleft classification systems and specific reasons why craniofacial specialists opt for these classification systems (Supplementary data file 1). The respondents were asked to specify the limitations of their preferred system and to identify the elements they believed were essential in the ideal cleft classification system. The questionnaire was designed and distributed using SurveyMonkey, an online survey application. Responses were gathered anonymously. A reminder was sent 14 days after the initial invitation.

The Strengthening the Reporting of Observational Studies in Epidemiology (STROBE) checklist was adhered to while preparing this manuscript. Numbers and percentages are obtained from SurveyMonkey and bar charts were designed using SPSS (Statistical Program for Social Sciences, version 26; SPSS Inc., Chicago, IL, USA) statistical software. Approval of the Institutional Review Board was received.

## Results

**Figure 1.**
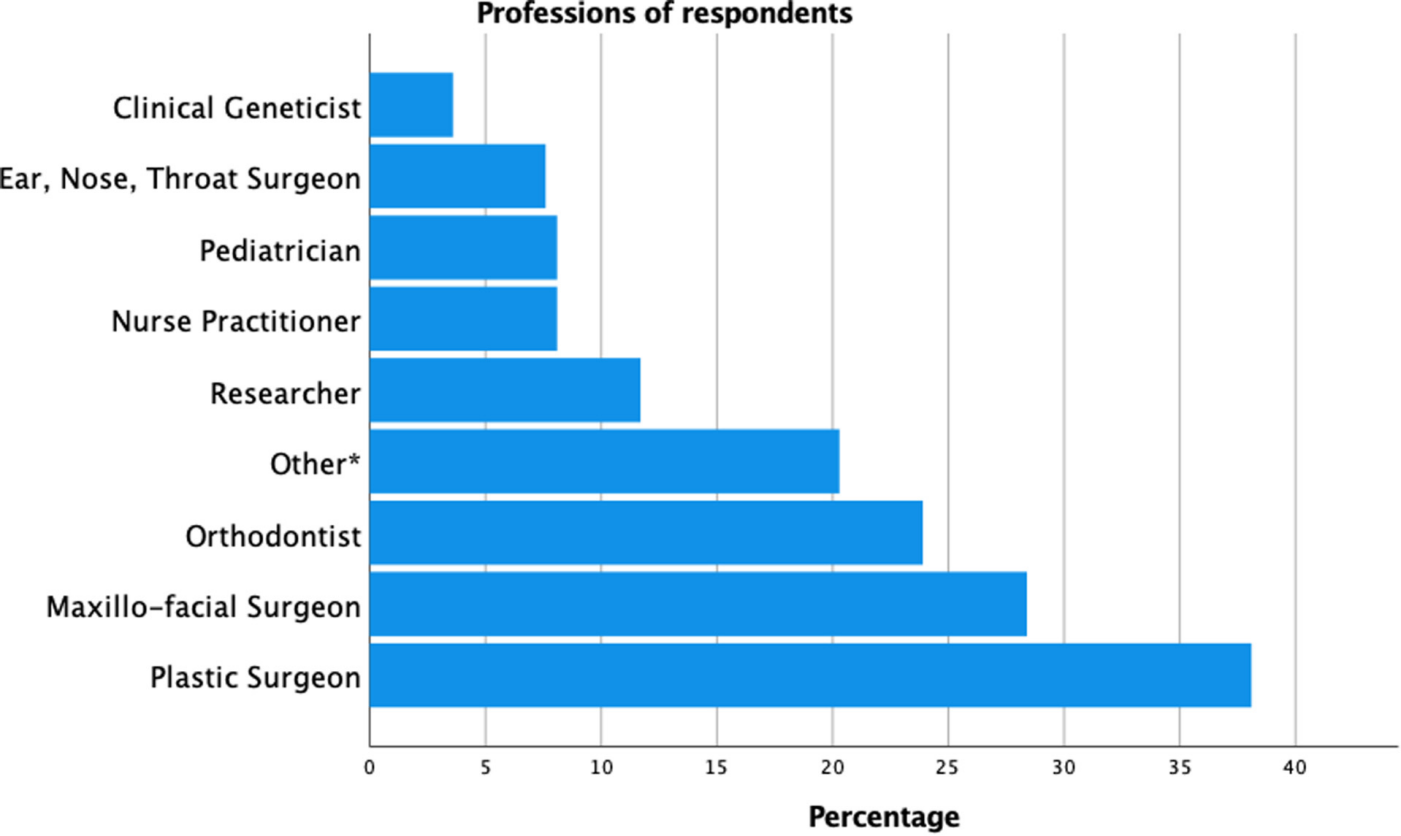
Distribution of professions among respondents. ^†^ Note that the sum of the number of professions is greater than 197 and subsequently exceeds 100%. The reason for this is that quite often respondents answered the survey on behalf of their entire cleft care team and filled in multiple professions.

A total of 197 respondents from 166 cleft centers and 61 countries completed the questionnaire between September 1, 2020 and October 31, 2020. Most respondents originated from Europe (50.0%), Asia (21.8%) and North America (13.2%) (Supplementary data file 2). Respondents included providers from a range of functions with the most frequent being plastic surgeons (38.1%), maxillofacial surgeons (28.4%), and orthodontists (23.9%) (Figure 1).

**Figure 2.**
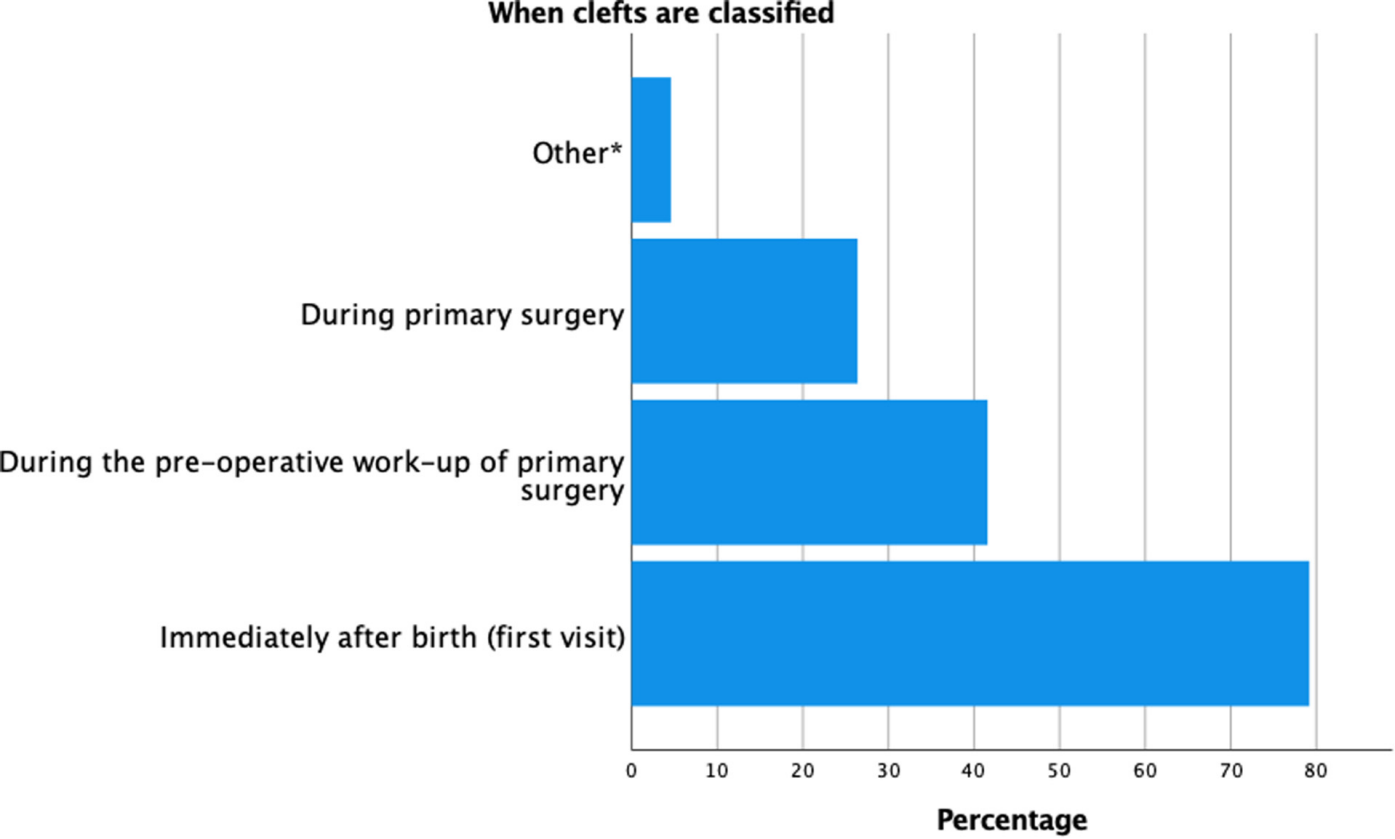
Timing of classifying clefts among respondents.

*Abbreviations*: *SABG; secondary alveolar bone grafting*.

The majority of respondents noted that the registration/classification was done multiple times during cleft management. Seventy-nine percent of the respondents classified clefts at the first visit, 41.6% during the pre-operative work-up, and 26.4% re-evaluated the classification during primary surgery (Figure 2). Fifty-four percent of the respondents noted that the classification is mainly performed by a specific specialist, primarily by plastic surgeons, maxillofacial surgeons or cleft surgeons. Furthermore, according to 81.2% of respondents, classification was done for both clinical and research purposes. Forty-two percent of the respondents stated that they also classify clefts for a national registration and 25.4% classified for reimbursement purposes.

**Figure 3.**
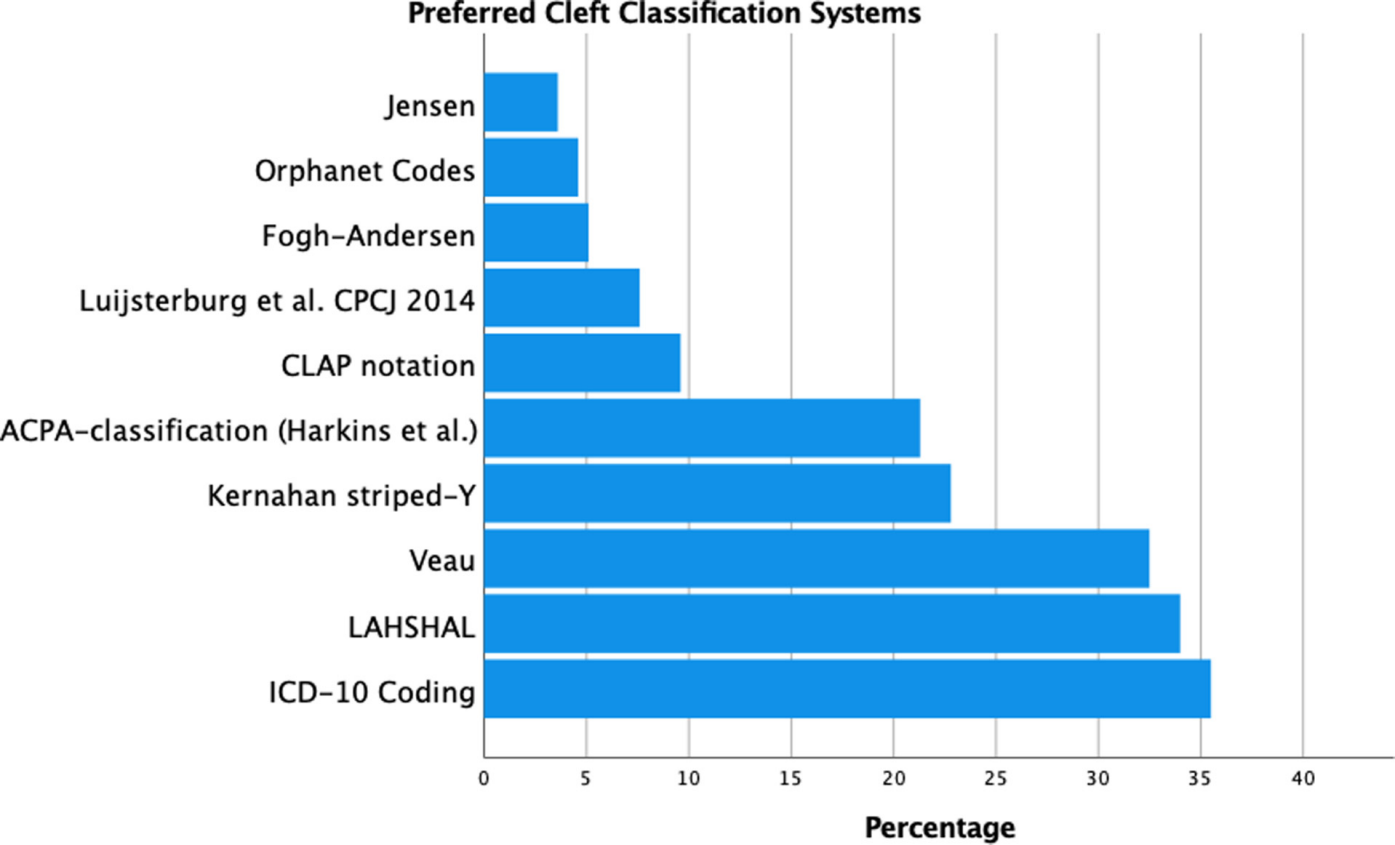
Preferred classification systems among respondents. It was possible for the participants to choose multiple systems. Abbreviations: ACPA; American Cleft Palate-Craniofacial Association; ICD-10: International Statistical Classification of Diseases and Related Health Problems, 10^th^ revision.

The most frequently employed classification systems were ICD-10 (35.5%), followed by LAHSHAL (34%), Veau (32.5%), Kernahan's striped-Y (22.8%) and the ACPA classification (Harkins et al., 1962) (21.3%) (Figure 3). A great variety existed in which classification systems were preferred both nationally and internationally, as shown in Table 1. Respondents from the United Kingdom were most homogenous in classifying clefts: 93.3% (14/15) respondents from the UK stated that they used LAHSHAL, since this was required for their national registry (CRANE, 2021). Multiple respondents reported that ICD-10 was required for reimbursement and registration purposes. Besides the ten classification systems proposed in our survey, respondents also indicated that another eight classification systems were used (Supplementary data file 3).

**Table 1.** Systems Used in Countries With Five or More Respondents. Expressed in Number of Respondents.^a^.

	LAHSHAL	Veau	Fogh-Andersen	Kernahan's striped-Y	ACPA (Harkins et al.)	CLAP notation	ICD-10	Orphanet codes	Jensen	Luijsterburg et al., CPCJ 2014
India (*n* = 24)	3	5	1	8	10	2	1	.	1	2
USA (*n* = 19)	3	13	.	1	8	5	13	.	1	.
UK (*n* = 15)	14	1	.	.	1	1	1	.	.	1
Germany (*n* = 10)	8	.	1	.	.	.	6	1	.	.
Netherlands (*n* = 10)	1	4	.	1	1	1	5	.	.	7
Sweden (*n* = 9)	.	3	1	2	.	.	6	1	.	.
Indonesia (*n* = 8)	3	2	.	1	.	2	4	.	.	1
Switzerland (*n* = 6)	6	.	1	.	1	.	.	1	.	.
Egypt (*n* = 5)	1	.	.	4	1	.	.	.	.	.
France (*n* = 5)	.	3	.	.	.	.	2	3	.	1

*Abbreviations: ACPA: American Cleft Palate-Craniofacial Association; CLAP: cleft lip, alveolus and palate; CPCJ: Cleft Palate-Craniofacial Journal; ICD-10: International Statistical Classification of Diseases and Related Health Problems, 10^th^ revision; LAHSHAL: lip, alveolus, hard palate, soft palate, hard palate, alveolus, lip*.

^a^ Note that several participants responded to this question with multiple classification systems. Therefore, the total of each column exceeds the participants per country.

When asked which information was most essential when using a preferred cleft classification, 32.5% of the respondents indicated that anatomical and morphological characteristics were essential in the classification process (Table 2). Furthermore, almost 20% of the respondents stated that the extension and severity of the cleft were decisive for their preferred system. These variables were essential since they influence both the timing and choice of treatment, according to 26% of the respondents. The differentiation of incomplete versus complete clefts, submucous clefts and whether the alveolus was affected, were also of importance for planning suitable treatment. Other applications for classification were research, clear documentation and registration, and the evaluation of treatment results.

**Table 2.** Essential Data and Manners in Which Way This Data is Essential According to Respondents.

Essential data	*N*
Anatomical and morphological characteristics	64
Severity/extension of cleft	39
The data influences timing and choice of treatment and surgery	52
The data is important for research purposes	23
The data is essential for documentation/registration	14
The data is essential for evaluating treatment results	9

When asked to identify limitations of their current cleft classification system, a frequent limitation of the previously mentioned classification systems was the imprecise description of the anatomical and morphological cleft characteristics (ie the severity and extension of the cleft) (Table 3). Furthermore, commonly used systems often did not include submucous clefts, Simonart's bands and nasal deformities. Another limitation cited was the fact that some clefts also failed to fit precisely into one of the categories of the used system. Due to these previously mentioned limitations, 8.6% (*n*  =  17) of respondents would not recommend their used classification system to other cleft caregivers.

**Table 3.** Recurring Limitations per Classification System. The Numbers Represent the Number of Respondents Addressing a Limitation to a Particular System.

	Anatomical description is too imprecise	SMCP not included	Lack of graphic information	Cleft does not fall neatly in categories	Too elaborate
LAHSHAL	21	7	1	1	.
Veau	24	3	2	1	.
Fogh-Andersen	2	.	.	.	.
Kernahan's striped-Y	12	2	.	.	.
ACPA	13	.	1	1	1
CLAP	5	.	1	.	.
Jensen	1	.	.	1	.
ICD-10	21	.	1	.	.
Orphanet Codes	2	.	.	1	.
Luijsterburg	1	.	.	1	1

Abbreviations: ACPA: American Cleft Palate-Craniofacial Association; CLAP: cleft lip, alveolus and palate; ICD-10: International Statistical Classification of Diseases and Related Health Problems, 10^th^ revision; LAHSHAL: lip, alveolus, hard palate, soft palate, hard palate, alveolus, lip; SMCP: submucous cleft palate.

An ideal classification should have a more precise description of the severity and extension of the cleft, according to 71 respondents (36.2%). Another important feature of an ideal classification is that the classification system is easy to use, which was mentioned by 43 respondents (21.8%). Twenty-one (34.4%) countries of a total of 61 countries within our respondents have a national registry for OFCs (Supplementary data file 4). In the United States of America, registry is state-dependent. Furthermore, 19.3% did not use a local or hospital registry.

## Discussion

The present study demonstrates great heterogeneity among clinicians involved in cleft care when they classify OFCs. A total of 197 clinicians from 166 centers around the globe completed the questionnaire. Respondents used eighteen different cleft registration or classification systems. Frequently cited limitations of existing systems were imprecise description of cleft extension and severity, failure to include submucous cleft palate, and difficulty in precisely classifying all clefts within the provided categories of a system.

As is mentioned in the introduction of this article, it is important to make a difference between registration and classification of clefts. Since systems that only register or register and classify are both commonly used for seemingly the same purposes, we did not distinguish between the two systems since we were only interested in the way OFC diagnoses were collected.

As respondents note, the anatomical description and the description of the extension or severity of the cleft are both the main components and greatest limitations at the same time for the majority of systems. For example, the Veau classification differentiates between the soft and hard palate but does not take into account cleft lip/alveolus and the morphological severity of palatal clefts (Veau, 1932). The ICD-10 system has been greatly improved compared to the ICD-9 system concerning the extensiveness of the anatomical varieties of clefts, but it does not allow for the documentation of morphological severity [i.e. completeness], laterality, the presence of asymmetry in the case of bilateral cleft lip/alveolus, microforms, or for clefts of the alveolus [Wang et al., 2014; Allori et al., 2016; McBride et al., 2016]. A system which solves at least one of these problems, the laterality problem, is the Kernahan striped-Y classification system [Kernahan, 1971]. Though, it cannot be used for verbal communication or description in the text format nor can it be used for computer archiving [Kernahan, 1973]. It was commonly used in the era of paper-based medical records, but its usage seems to have has diminished considerably with the advent of electronic health records [EHRs].

Kriens made some valuable suggestions about an ideal classification system. He stated that the following were pre-requisites in an ideal recording system or index for CLP: it should be: 1. Simple, so as to be accepted. 2. Concise, for accurate recording. 3. Flexible, to account for even rare presentations. 4. Exact, to facilitate research and statistics. 5. Morphological, to be able to visually assess. 6. Graphic, for clarity and to avoid ambiguity (Kriens, 1989). In an attempt to reach this ideal classification system, Kriens developed the LAHSHAL system.

LAHSHAL is a palindrome which is based upon the anatomy of the patient and proceeds from the right side towards the left side, designed to facilitate the recording during clinical examination. Each column of the acronym is filled as follows to confirm whether that part of the anatomy is involved. A capital letter is noted when an anatomic structure is completely clefted while a lowercase letter is noted when the cleft is only incomplete. Furthermore, some symbols can be used to describe other deviations. Namely, an asterisk (*) is noted when minimal clefting (eg, lesser-form cleft lip, notched alveolus, submucous cleft palate) occurs, and a dot (•/.) means that the anatomic feature is unaffected. A plus sign can be noted in the L column when a skin band (ie a Simonart's band) is present. Note in table 3 that a number of participants indicated that the LAHSHAL lacks anatomic specificity. However, not all clinicians are fully aware of the potential of this extremely elaborate classification system. Due to its extensiveness, over 12 000 combinations can be made when using the LAHSHAL system for OFCs. As such, it is extremely detailed but as a result of this it has a steep learning curve to implement it in clinical practice. Finally, the LAHSHAL system lends it well to use in electronic health records.

McBride et al., have suggested that ideally, a coding system for OFCs should be precise, logical, reproducible, accurate, and sufficiently descriptive (McBride et al., 2016). They suggest that it must be precise enough to differentiate between subphenotypes; it should be user-friendly and accurate to be used globally between various health care professionals and epidemiologists with little need for training. It should also be sufficiently descriptive to differentiate laterality and completeness to facilitate the future of genetic and etiological research while being easily interpreted by users from different research and clinical backgrounds. In addition to these requirements, the coding system must remain relevant to the current globally recognized system of recording disease, the ICD-10 system. McBride et al., illustrated how LAHSHAL codes could be converted to ICD-10, and potentially be automated using an algorithm (McBride et al., 2016). Therefore, the LAHSHAL classification (or a variant) and the Luijsterburg et al., classification, as described in this manuscript, are potentially useful tools and could be a complement to the ICD-10 system.

Several limitations of this study have to be addressed. A total of 197 respondents among 2140 invitations completed our survey. Some healthcare professionals received multiple invitations when they attended more than one congress. It was not clearly mentioned whether a respondent had to respond individually or on behalf of their entire cleft team. Consequently, the response rate is unknown. Furthermore, participation bias is inseparable with cross-sectional survey studies, which hinders making global recommendations. This survey demonstrated that LAHSHAL and Luijsterburg et al., were most used. Since the usage of Luijsterburg et al., is limited to Europe, we recommend LAHSHAL as optimal classification system. Another limitation of the survey is that no distinction was made between state, regional registries and national registries. For example, in the USA, some states have state registries although a national registry does not exist. Therefore, we excluded the USA from this specific question. Moreover, this questionnaire involved only clinicians involved in the care of patients with OFCs. Policy makers, hospital staff members involved in the financial reimbursement and insurance companies were not involved in the survey despite their role in the cleft care process.

## Conclusions

Great variety of classification systems currently used exists among clinicians when confronted with patients with OFCs. Because future advances in our understanding of the molecular pathogenesis of OFCs will require us to compare precisely phenotyped cohorts of patients with clefts, further research and involvement among geneticists and embryologists is needed to provide optimal comprehension of classifying clefts amongst all craniofacial specialists. Classifying clefts with a universally implemented classification system is essential for improved communication between craniofacial specialists and comparison of scientific data globally. According to the results of this study, LAHSHAL could be the most suited universal classification system, due to its extensiveness, relatively high implementation rate and convenient usage to complement the ICD-10 system. Potentially, this could make up for the deficiencies of the ICD-10 codes that do not indicate laterality, completeness, and clefts of the alveolus. For more international acceptance, standardization and better communication globally it is prudent to demonstrate to users the merits of the LAHSHAL classification system.

## Supplemental Material

sj-docx-1-cpc-10.1177_10556656211057368 - Supplemental material for Classification Systems of Cleft Lip, Alveolus and Palate: Results of an International Survey Click here for additional data file. Supplemental material, sj-docx-1-cpc-10.1177_10556656211057368 for Classification Systems of Cleft Lip, Alveolus and Palate: Results of an International Survey by Ruben Houkes, Johannes Smit and Peter Mossey, Peter Don Griot, Martin Persson, Amanda Neville, Edwin Ongkosuwito, Tom Sitzman, Corstiaan Breugem in The Cleft Palate Craniofacial Journal

sj-docx-2-cpc-10.1177_10556656211057368 - Supplemental material for Classification Systems of Cleft Lip, Alveolus and Palate: Results of an International Survey Click here for additional data file. Supplemental material, sj-docx-2-cpc-10.1177_10556656211057368 for Classification Systems of Cleft Lip, Alveolus and Palate: Results of an International Survey by Ruben Houkes, Johannes Smit and Peter Mossey, Peter Don Griot, Martin Persson, Amanda Neville, Edwin Ongkosuwito, Tom Sitzman, Corstiaan Breugem in The Cleft Palate Craniofacial Journal

sj-docx-3-cpc-10.1177_10556656211057368 - Supplemental material for Classification Systems of Cleft Lip, Alveolus and Palate: Results of an International Survey Click here for additional data file. Supplemental material, sj-docx-3-cpc-10.1177_10556656211057368 for Classification Systems of Cleft Lip, Alveolus and Palate: Results of an International Survey by Ruben Houkes, Johannes Smit and Peter Mossey, Peter Don Griot, Martin Persson, Amanda Neville, Edwin Ongkosuwito, Tom Sitzman, Corstiaan Breugem in The Cleft Palate Craniofacial Journal

sj-docx-4-cpc-10.1177_10556656211057368 - Supplemental material for Classification Systems of Cleft Lip, Alveolus and Palate: Results of an International Survey Click here for additional data file. Supplemental material, sj-docx-4-cpc-10.1177_10556656211057368 for Classification Systems of Cleft Lip, Alveolus and Palate: Results of an International Survey by Ruben Houkes, Johannes Smit and Peter Mossey, Peter Don Griot, Martin Persson, Amanda Neville, Edwin Ongkosuwito, Tom Sitzman, Corstiaan Breugem in The Cleft Palate Craniofacial Journal
