# Supplementary material for: Classification Systems of Cleft Lip, Alveolus and Palate: Results of an International Survey
Source: Cleft Palate Craniofac J. 2021 Nov 23;60(2):189–96. doi: 10.1177/10556656211057368 (PMC9843539; doi:10.1177/10556656211057368)
Supplement: sj-docx-1-cpc-10.1177_10556656211057368 - Supplemental material for Classification Systems of Cleft Lip, Alveolus and Palate: Results of an International Survey [file sj-docx-1-cpc-10.1177_10556656211057368.docx]

**Supplementary data 1:** Questionnaire

1. **Hospital name**
2. **City**
3. **Country**
4. **Function (check all that apply)**

- Pediatrician
- Clinical Geneticist
- Plastic Surgeon
- Maxillofacial Surgeon
- ENT Surgeon
- Orthodontist
- Nurse Practitioner
- Researcher
- Other

1. **Do you have a national cleft registration (i.e. do you report clefts to a state or national registry?)**

- Yes
- No

1. **Do you have a cleft registration for your local hospital or cleft team?**

- Yes
- No

1. **When do you classify clefts? (Check all that apply)**

- Immediately after birth (first visit)
- During the pre-operative work-up of the primary surgery
- During the primary surgery
- Other

1. **Who records the classification of orofacial clefts? (Check all that apply)**

- The specialist who first suspected the possibility of a cleft anomaly and performed diagnostic tests accordingly
- A specific specialist (for example: Pediatrician, surgeon, geneticist etc.)
- Through a multidisciplinary meeting
- Other

1. **In case you answered the last question with 'a specific specialist', which specialist records at your team/institution the classification of clefts?
   (In case you did not answer the last question with 'a specific specialist', you can skip this question)**
2. **Why are clefts being classified in your institution? (Check all that apply)**

- For national registration
- For reimbursement
- For embryological purposes
- For clinical and fundamental research
- Other

1. **Which classification system do you/your team currently use for the classification of clefts immediately after birth? Please make a selection of the different classification systems listed below, multiple answers are possible. Please specify if you use another system.**

- LAHSHAL (Kriens, 1989)
- Veau (Veau, 1932)
- Fogh-Andersen (Fogh-Andersen, 1971)
- Kernahan’s striped-Y (Kernahan, 1971)
- ACPA classification (Harkins et al., 1962)
- CLAP notation (Allori et al., 2017)
- Jensen (Jensen et al., 1988)
- ICD-10 coding (World Health Organization, 2021c)
- Orphanet Codes (Orphanet, 2021)
- Luijsterburg et al. CPCJ 2014 (Luijsterburg et al., 2014)

1. **In the case you missed a system in the previous question, please name, specify and describe it in the box below**
2. **In the case you use multiple systems for classifying clefts, do you use these in a particular order? If so, describe this particular order and why you use it this way.**
3. **Please rate in what capacity the reasons mentioned below are applicable to your preferred classification system**

|  | **Strongly disagree** | **Disagree** | **Neutral** | **Agree** | **Strongly agree** |
| --- | --- | --- | --- | --- | --- |
| **It is fast** |  |  |  |  |  |
| **It is easy to use** |  |  |  |  |  |
| **Anatomical and morphological CLAP notation is required** |  |  |  |  |  |
| **Unsure** |  |  |  |  |  |

If another reason for using your preferred system is applicable but not mentioned above, please do so here and specify

1. **Which information/data is essential in your line of work for using your preferred classification system and why is this information essential?**
2. **What are the limitations of your preferred system, chosen in the previous questions, and why do these limit the system?**
3. **Would you recommend your preferred system to other cleft caregivers?**

- Yes
- No

1. **What should your ideal classification system consist of and why?**
2. **If you have any other comments regarding cleft classification systems which hasn’t been addressed in the questionnaire, you can comment them below**
3. **In the future we would like to conduct a consensus meeting. Do you allow us to contact you for any possible future international events/meetings/conferences regarding the classification of clefts?**

- Yes
- No
