# Supplementary material for: Classification Systems of Cleft Lip, Alveolus and Palate: Results of an International Survey
Source: Cleft Palate Craniofac J. 2021 Nov 23;60(2):189–96. doi: 10.1177/10556656211057368 (PMC9843539; doi:10.1177/10556656211057368)
Supplement: sj-docx-2-cpc-10.1177_10556656211057368 - Supplemental material for Classification Systems of Cleft Lip, Alveolus and Palate: Results of an International Survey [file sj-docx-2-cpc-10.1177_10556656211057368.docx]

**Supplementary data 2.** Country of origin of respondents with number of respondents from

each country.

| **Europe**  (n=99) | **Asia**  (n=43) | **Middle-East**  (n=15) | **North America** (n=22) | **South America** (n=8) | **Middle America** (n=4) | **Africa**  (n=4) | **Oceania**  (n=3) |
| --- | --- | --- | --- | --- | --- | --- | --- |
| Austria (2) | Bangladesh (2) | Afghanistan (1) | Canada (3) | Argentina (2) | Mexico (3) | Ethiopia (2) | Australia (2) |
| Belgium (2) | India (24) | Bahrain (1) | United States of America (19) | Brazil (2) | Puerto Rico (1) | Kenya (1) | New Zealand (1) |
| Bulgaria (1) | Indonesia (8) | Egypt (5) |  | Chile (2) |  | Madagascar (1) |  |
| Croatia (3) | Japan (1) | Israel (2) |  | Colombia (1) |  |  |  |
| Cyprus (1) | Malaysia (1) | Pakistan (1) |  | Peru (1) |  |  |  |
| Czech Republic (2) | Myanmar (1) | Saudi Arabia (1) |  |  |  |  |  |
| Denmark (1) | Nepal (1) | Turkey (4) |  |  |  |  |  |
| Estonia (2) | Philippines (3) |  |  |  |  |  |  |
| Finland (1) | Sri Lanka (2) |  |  |  |  |  |  |
| France (5) |  |  |  |  |  |  |  |
| Germany (10) |  |  |  |  |  |  |  |
| Greece (2) |  |  |  |  |  |  |  |
| Italy (3) |  |  |  |  |  |  |  |
| Latvia (4) |  |  |  |  |  |  |  |
| Lithuania (2) |  |  |  |  |  |  |  |
| Montenegro (1) |  |  |  |  |  |  |  |
| Netherlands (10) |  |  |  |  |  |  |  |
| Norway (2) |  |  |  |  |  |  |  |
| Poland (1) |  |  |  |  |  |  |  |
| Portugal (1) |  |  |  |  |  |  |  |
| Republic of Ireland (1) |  |  |  |  |  |  |  |
| Republic of North Macedonia (1) |  |  |  |  |  |  |  |
| Romania (1) |  |  |  |  |  |  |  |
| Russia (3) |  |  |  |  |  |  |  |
| Serbia (2) |  |  |  |  |  |  |  |
| Slovak Republic (1) |  |  |  |  |  |  |  |
| Slovenia (1) |  |  |  |  |  |  |  |
| Spain (3) |  |  |  |  |  |  |  |
| Sweden (9) |  |  |  |  |  |  |  |
| Switzerland (6) |  |  |  |  |  |  |  |
| United Kingdom (15) |  |  |  |  |  |  |  |
