# Supplementary material for: Classification Systems of Cleft Lip, Alveolus and Palate: Results of an International Survey
Source: Cleft Palate Craniofac J. 2021 Nov 23;60(2):189–96. doi: 10.1177/10556656211057368 (PMC9843539; doi:10.1177/10556656211057368)
Supplement: sj-docx-3-cpc-10.1177_10556656211057368 - Supplemental material for Classification Systems of Cleft Lip, Alveolus and Palate: Results of an International Survey [file sj-docx-3-cpc-10.1177_10556656211057368.docx]

**Supplementary data 3.** Other classification systems that were mentioned being used by the respondents.

| SNOMED CT (SNOMED International, 2021) |
| --- |
| Bangalore Classification System (Subramani et al., 2005) |
| International cleft classification (Wang et al., 2014) |
| Indian classification of cleft lip and palate. (Balakrishnan, 1975) |
| Elsherbiny’s cleft classification system (Elsherbiny et al., 2017) |
| Registration system of the Dutch Association for Cleft Palate and Craniofacial Anomalies. (Luijsterburg et al., 2011) |
| The Clock Diagram. (Rossel-Perry, 2009) |
| LAHSN system. (Koch et al., 1995) |

Abbreviations: LAHSN: lip, alveolus, hard and soft palate, nose; SNOMED CT: SNOMED Clinical Terms.
