# Supplementary material for: Classification Systems of Cleft Lip, Alveolus and Palate: Results of an International Survey
Source: Cleft Palate Craniofac J. 2021 Nov 23;60(2):189–96. doi: 10.1177/10556656211057368 (PMC9843539; doi:10.1177/10556656211057368)
Supplement: sj-docx-4-cpc-10.1177_10556656211057368 - Supplemental material for Classification Systems of Cleft Lip, Alveolus and Palate: Results of an International Survey [file sj-docx-4-cpc-10.1177_10556656211057368.docx]

**Supplementary data 4.** Presence of a national registry per country according to the respondents.^a^

| **National registry**  n = 21 (34.4%) | **No national registry**  n = 39 (63.9%)^b^ |
| --- | --- |
| Afghanistan | Australia |
| Argentina | Austria |
| Bulgaria | Bahrain |
| Chile | Bangladesh |
| Cyprus | Belgium |
| Czech Republic | Brazil |
| Finland | Canada |
| France | Colombia |
| Republic Ireland | Croatia |
| Latvia | Denmark |
| Netherlands | Egypt |
| New Zealand | Estonia |
| Norway | Ethiopia |
| Portugal | Germany |
| Puerto Rico | Greece |
| Saudi Arabia | India |
| Slovak Republic | Indonesia |
| Slovenia | Israel |
| Sweden | Italy |
| Switzerland | Japan |
| United Kingdom | Kenya |
|  | Lithuania |
|  | Madagascar |
|  | Malaysia |
|  | Mexico |
|  | Montenegro |
|  | Myanmar |
|  | Nepal |
|  | Republic of North Macedonia |
|  | Pakistan |
|  | Peru |
|  | Philippines |
|  | Poland |
|  | Romania |
|  | Russia |
|  | Serbia |
|  | Sri Lanka |
|  | Spain |
|  | Turkey |

^a^ Registry in USA is state-dependent. We therefore excluded the USA from this analysis.

^b^ Note that the total number of countries does not reach 100% due to exclusion of the USA.
